# Experience With Fenestrated Endovascular Repair of Juxtarenal Abdominal Aortic Aneurysms at a Single Center

**DOI:** 10.1097/MD.0000000000002683

**Published:** 2016-03-11

**Authors:** Zhongzhou Hu, Yue Li, Ran Peng, Jie Liu, Tao Zhang, Wei Guo

**Affiliations:** From the Medical Center Tsinghua University (ZH); Department of Vascular Surgery, General Hospital of People's Liberation Army (YL, JL, WG); State Key Laboratory of Microbial Technology, School of Life Science, Shandong University, Jinan 250100, China (RP); and Department of Vascular Surgery, Peking University People's Hospital, Beijing, China (TZ).

## Abstract

To present the early and mid-term results of fenestrated endovascular aneurysm repair (FEVAR) using the Zenith fenestrated device for juxtarenal abdominal aortic aneurysms (JAAAs) at our center in China.

Design: Retrospective study.

The study included 15 male patients with JAAAs, who underwent FEVAR using the Zenith fenestrated device at our center between February 2011 and June 2015.

All custom-made Zenith fenestrated devices were designed according to computed tomography angiography (CTA) images obtained preoperatively. The patients with renal insufficiency underwent duplex ultrasonography, while the patients with normal renal function underwent 3 CT data acquisitions including nonenhanced CT, arterial phase, and venous phase. These examinations and blood examinations were completed at 3, 6, and 12 months after discharge, and annually thereafter.

The mean age of the patients was 73.13 ± 9.06 years (range, 57–86 years), and the median follow-up period was 30 months (8–52 months). Small fenestrations were used in 27 renal arteries, scallops were used in 7 superior mesenteric arteries (SMAs) and 2 renal arteries, and large fenestrations were used in 2 SMAs. Conversion to an open procedure was not required in any of the patients, and the technical success rate was 100%. The mean length of hospital stay was 11.33 ± 2.02 days (7–15 days). No patient died within the 1st 30 days after the operation. One patient had a type Ia endoleak, which disappeared at 6 months after the operation, 1 patient had a type Ib endoleak, which was detected at 17 months after the operation, and 2 patients had type II endoleaks. One patient died at 17 months and another patient died at 30 months after the operation. Therefore, the all-cause mortality rate was 13.33% (2/15). The target vessel patency rate was 100% without occlusion.

The early and mid-term results of FEVAR using the Zenith fenestrated device were good, demonstrating that this procedure is effective for the treatment of JAAAs.

## INTRODUCTION

Endovascular aneurysm repair (EVAR) is a less invasive treatment for abdominal aortic aneurysms (AAAs),^1,2^ especially for patients with severe comorbidities. However, owing to differences in anatomical morphology, not all AAAs can be treated with conventional EVAR. Juxtarenal abdominal aortic aneurysms (JAAAs) are complex AAAs that have a short proximal aortic neck (proximal seal zone <15 mm). These aneurysms account for approximately 15% of all AAAs.^3^

Fenestrated grafts were 1st used as an endoluminal approach for patients with JAAAs in 1996.^4^ Over the past 19 years, fenestrated endovascular aneurysm repair (FEVAR) has been shown to be an effective treatment for JAAAs, with satisfactory outcomes.^5–7^ In 2011, the State Food and Drug Administration of China approved the Zenith fenestrated device (Cook Medical, Bloomington, IN) for commercial use in China. The 1st procedure using the Zenith fenestrated device in the mainland of China was performed at our center in February 2011, and since then, our center has performed 45% of the total number of procedures involving the use of the Zenith fenestrated device for JAAAs in the mainland of China.

Although there are reports about FEVAR from other centers with good short, mid-term outcomes, none reports presented the usage information of the Zenith fenestration device in China and the feasibility of this device for Chinese patients. The aim of the present study was to present the early and mid-term results of FEVAR using the Zenith fenestrated device for JAAAs at our center that has performed the maximum number of these procedures in the mainland of China.

## PATIENTS AND METHODS

### Study Population

This retrospective study included 15 male patients with JAAAs who underwent FEVAR using the Zenith fenestrated device at the General Hospital of People's Liberation Army, Beijing, China between February 2011 and June 2015. Patients with serious cardiovascular diseases, pulmonary comorbidities, or an American Society of Anesthesiologists score of 3 or more were considered at high risk for open surgery. In our series, all patients were at high risk for open surgery. The study was approved by the ethics committee of Chinese People's Liberation Army General Hospital and all patients signed informed consents.

### Preoperative Assessment

All patients underwent cardiac ultrasonography, chest radiography, and electrocardiography to assess cardiopulmonary function. CT was performed by using a 256-slice computed tomography scanner (iCT, Philips-Healthcare) with a 1-mm section thickness and 0.5-mm intervals. Preoperative computed tomography angiography (CTA) with axial and coronal reconstructions were performed to evaluate anatomical morphology and distance from the distal descending thoracic aorta to the profunda femoral artery. Detailed information on aneurysm diameter, aneurysm morphology, proximal and distal lengths, neck diameter, angulations, location, and ostial diameters of each visceral vessel were obtained, and the information was sent to the manufacturer of the Zenith fenestrated device.

The morphology of JAAAs for the use of the Zenith fenestrated device included the following: aneurysm diameter >50 mm; proximal neck angulation ≤45.0° relative to the long axis of the aneurysm; proximal neck diameter 19.0 to 31.0 mm; proximal neck length 4.0 to 15.0 mm; and access vessel that can accommodate a 20 Fr (8.0 mm outside diameter) sheath.

### Procedure

All procedures were performed in a hybrid operating room equipped with GE Innova 3100-IQ (GE Healthcare, Waukesha, WI). A proctor surgeon performed all procedures in this study. All patients underwent repair under general anesthesia, and heparin (80–100 U/kg) was administered after puncture of the bilateral femoral arteries.

**FIGURE 1:**
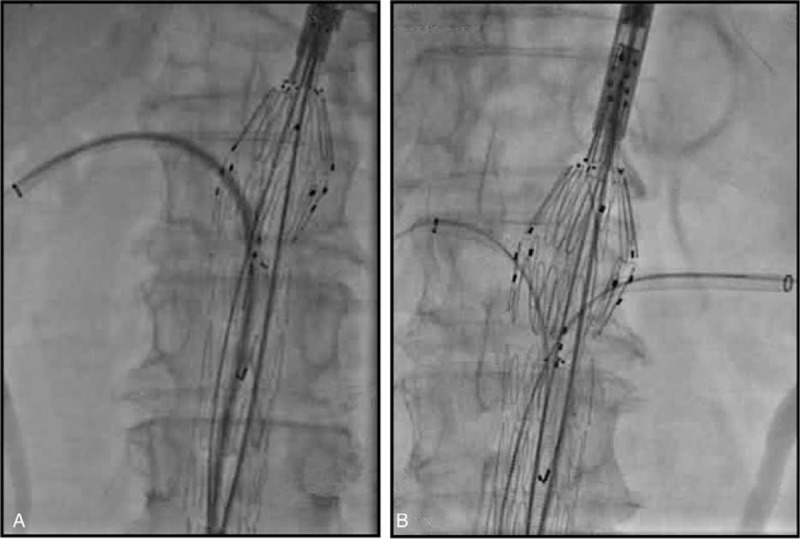
(A) Reconstruction for the right renal artery. (B) Reconstruction for the left renal artery.

**FIGURE 2:**
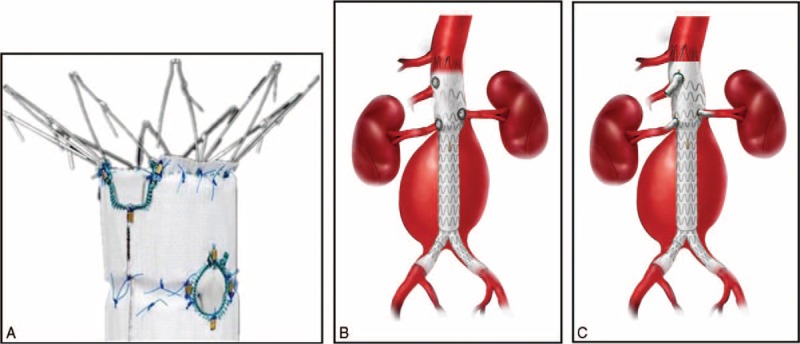
(A) The Zenith fenestrated device with fenestrations and scallops. (B) Illustration showing alignment of the fenestrations with the target vessels. (C) Illustration showing deployment of the stent in the target vessel.

The proximal fenestrated component was delivered from one of the femoral arteries, and it was introduced and oriented by visualizing radiopaque gold markers to target the fenestrations and/or scallops at the visceral vessels. A valved sheath (20–22F; Cook Medical) was used in the contralateral femoral vessel as access for target vessel sheaths and the angiographic catheter. The proximal component was partially deployed, and the fenestrations and/or scallops were aligned with the visceral vessels. Balloon-expandable covered stents (Joestent, Abbott, Temecula, California) were then deployed into the target arteries through the fenestrations, and the proximal component was completely released (Figure 1). A 10-mm balloon was used to achieve flaring of the proximal aortic end of the visceral vessel stent in order to ensure adequate seal and prevent migration. The placement of the distal bifurcated component was similar to that of the conventional bifurcated Zenith device, and at least 2 of its sections were overlapped with the proximal component. Finally, a compliant balloon was used to inflate all joints and sealing zones before completion angiography (Figure 2).

Completion angiography was performed to detect possible endoleaks, assess the patency of renal arteries, and check for aneurysm exclusion. Technical success was defined as the successful deployment of all stent grafts in the planned positions.

### Postoperative Management and Follow-Up

All patients received hydration therapy (1000 mL normal saline) in the 12 hours before the operation to the 1st 24 hours after FEVAR, and dual antiplatelet therapy was administered for the next 6 months (aspirin 100 mg and clopidogrel 75 mg). Moreover, life-long administration of single antiplatelet therapy was adopted. Serum creatinine levels were assessed before the operation, 3 days after the operation, and at discharge.

The patients with renal insufficiency underwent duplex ultrasonography. The patients with normal renal function underwent 3 CT data acquisitions including nonenhanced CT, arterial phase, and venous phase. The 120 to 150 mL iohexol 300 (Omnipaque 300; Sanofi-Winthrop, New York, NY) was administered intravenously at a rate of 4 mL/sec by using a power injector. Arterial phase CT data acquisition was initiated in the aorta at the level of the celiac artery reached 120 HU. Venous phase acquisition was initiated 60 seconds after arterial phase. All patients experienced blood examinations at 3, 6, and 12 months after discharge, and annually thereafter.

### Statistical Analysis

Continuous variables are presented as means ± standard deviations (SDs), and categorical variables are presented as percentages. Time-dependent outcomes were reported with Kaplan–Meier estimates. All statistical analyses were performed using SPSS version 20.0 for Windows (IBM, Armonk, NY).

## RESULTS

### Patient Characteristics

The study included 15 male patients with JAAAs who underwent FEVAR using the Zenith fenestrated device. The patient characteristics are presented in Tables 1 and 2. The mean age of the patients was 73.13 ± 9.06 years (range, 57–86 years). The mean aneurysm diameter was 68.07 ± 11.26 mm (range, 51–85 mm), and the mean angle of the proximal aneurysmal neck was 33° ± 18.96° (range, 0°–60°). The mean length of the infrarenal aortic neck was 5.6 ± 2.82 mm (range, 0–9 mm).

**TABLE 1:**
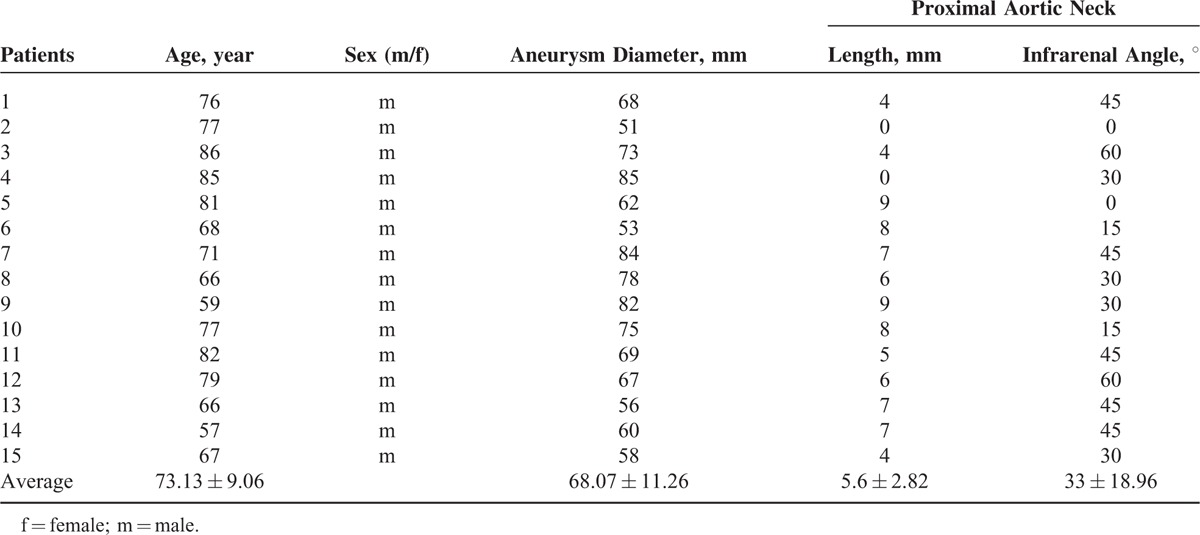
Patient Characteristics

**TABLE 2:**
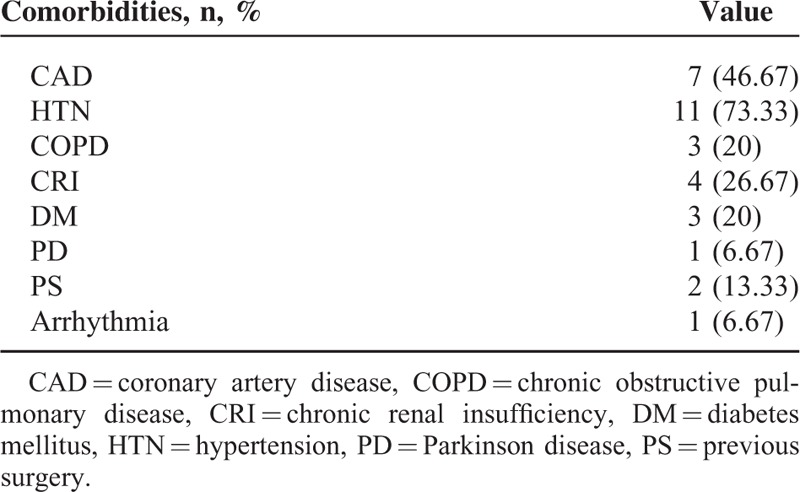
Preoperative Comorbidity

### Device Design

FEVAR was performed for 29 renal arteries (15 left and 14 right) and 9 superior mesenteric arteries (SMAs) (Table 2). Small fenestrations were used in 27 renal arteries, scallops were used in 7 SMAs and 2 renal arteries, and large fenestrations were used in 2 SMAs. Among the 15 patients, 7 (46.67%) were treated with a combination of small fenestrations for 2 renal arteries and a scallop for the SMA, 2 (13.33%) were treated with a combination of small fenestrations for 2 renal arteries and a large fenestration for the SMA, 4 (26.67%) were treated with small fenestrations for 2 renal arteries, 1 (6.67%) was treated with small fenestrations for 1 renal artery, and 1 (6.67%) was treated with scallops for 2 renal arteries.

### Procedural Results

The procedure was successfully performed in all patients, and the technical success rate was 100%. The mean operation time was 238.33 ± 55.35 minutes (range, 170–360 minutes), and the median blood loss was 400 mL (range, 100–1000 mL). The mean fluoroscopy time was 85.67 ± 27.77 minutes (range, 60–160 minutes), and the mean amount of contrast medium used was 303.33 ± 56.27 mL (range, 250–450 mL). The mean length of hospital stay was 11.33 ± 2.02 days (range, 7–15 days) (Table 3).

**TABLE 3:**
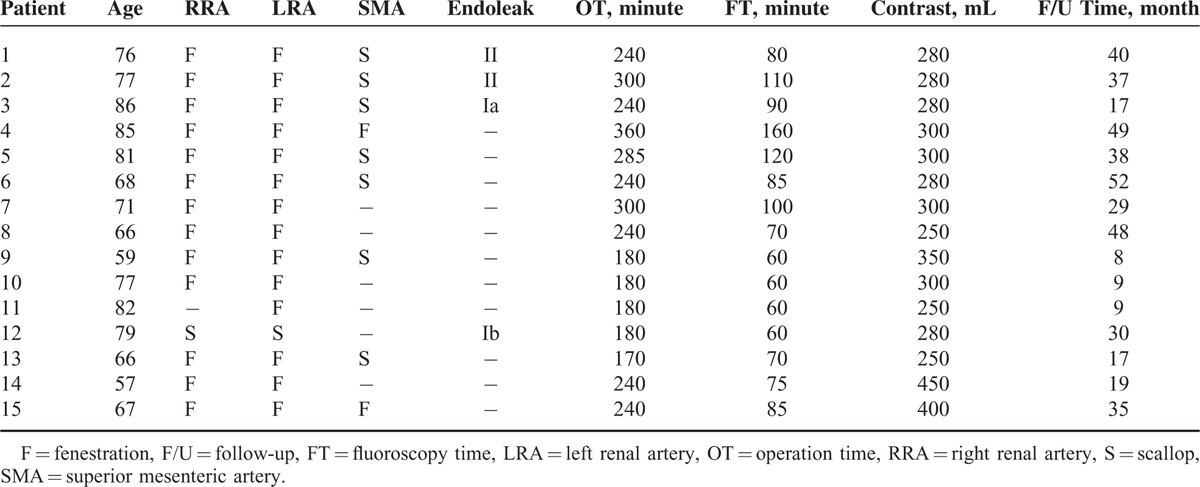
Procedural Data for Each Patient

The main body endografts were all custom-made Zenith fenestrated devices. A total of 29 balloon-expandable covered stents (Joestent, Abbott) were deployed into 29 visceral arteries (14 left renal arteries, 13 right renal arteries, and 2 SMAs) to secure the fenestrations. Stent grafts were successfully deployed in the planed positions in all cases.

### Endoleaks and Secondary Interventions

To the latest follow-up, there were no endoleaks occurring in all of 4 patients with renal insufficiency. Type I endoleaks occurred in 2 patients. One patient had a type Ia endoleak, which was detected on complete angiography; however, the patient did not require secondary intervention as the endoleak disappeared at 6 months after the operation. The other patient had a type Ib endoleak that was detected during the arterial phase of enhancement and caused a right common iliac aneurysm (CIA), which was detected on CTA at 17 months after the operation. The condition was resolved with internal iliac artery (IIA) embolization and insertion of 2 overlapped Fluency stents (Bard Inc, Karlsruhe, Germany) into the gap between the iliac extension stent graft and the aneurismal wall and 1 Express LD stent (Boston Scientific, Natick, MA) for extending the iliac extension stent graft. Type II endoleaks were identified in 2 patients on complete angiography. Latest CTA showed these 2 type II endoleaks still existed both during the venous phase of enhancement. However, the aneurysms in these patients increased by less than 5 mm; therefore, both patients received conservative therapy with regular imaging surveillance (Table 3). Among the other 11 patients, the aneurysm diameter reduced in 9 patients and was stable in 2 patients.

The left CIA ruptured in 1 patient at 30 months after the operation. In this patient, the left IIA was coiled, and then, 2 overlapped Endurant balloon-expandable stents (Medtronic Inc., Sunrise, FL) were inserted to extend the iliac extension stentgraft. Additionally, stenosis of the right iliac extension stent graft was noted, and it was treated with an Express LD stent. Among the 15 patients, 2 (13.33%) required secondary interventions.

### Mortality and Morbidity

**FIGURE 3:**
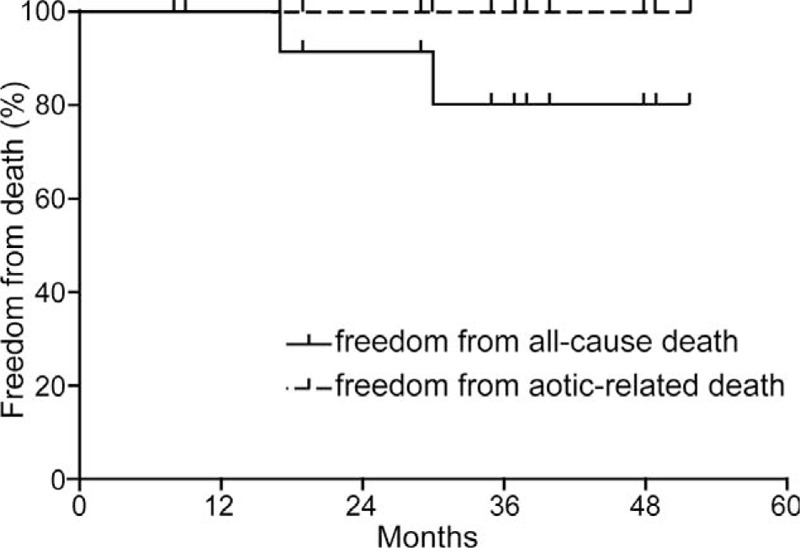
Kaplan–Meier analysis of freedom from death.

All 15 patients were followed up. The median follow-up time was 30 months (range, 8–52 months). The 30-day postoperative mortality rate was 0%, and the all-cause mortality rate was 13.33% (2/15). One patient died from hemispheric stroke at 30 months after the operation. The other patient died from a condition that was unrelated to the aorta at 17 months after the operation, and the patient's family members were unwilling to provide more information about the cause of death (Figure 3).

One patient experienced left renal artery dissection at the distal covered stent intraoperatively and was treated with a Zilver stent (Cook Medical) combined with a Scuba stent. The definition of contrast-induced nephropathy (CIN) is an increase of 25% or more, or an absolute increase of 0.5 mg/dL or more in serum creatinine from baseline value, at 48 to 72 hours following the exposure to contrast media. According to this definition, 2 patients with renal insufficiency experienced CIN after FEVAR. In these 2 patients, the postoperative creatinine level increased, respectively, 50 and 65 mol/L compared with preoperative level, and decreased and stabilized at the preoperative baseline, respectively, at 7 and 10 days after the operation. One patient each had arrhythmia, heart failure, pneumonia, urinary infection, and puncture point hematoma. All of these complications were treated conservatively, and they did not result in serious consequences.

**FIGURE 4:**
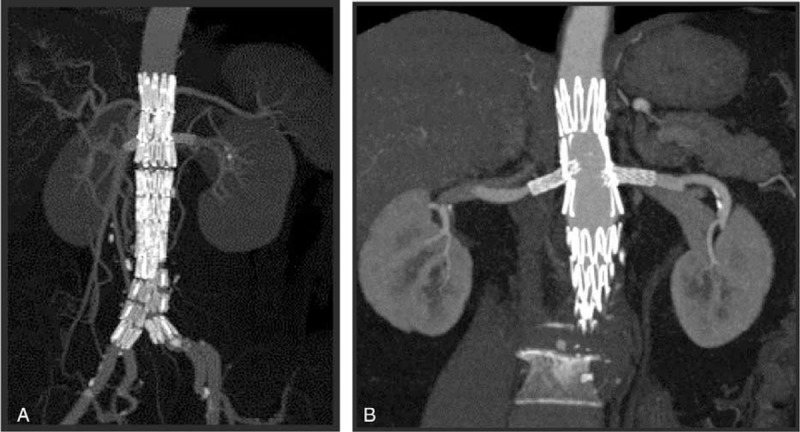
(A, B) Two computed tomography angiography images showing patency of 2 renal arteries without endoleaks and endograft migration at 1 year after the operation.

The target visceral branch patency rate was 100% during the follow-up period, and no migration or fracture of the devices was noted (Table 4; Figure 4).

**TABLE 4:**
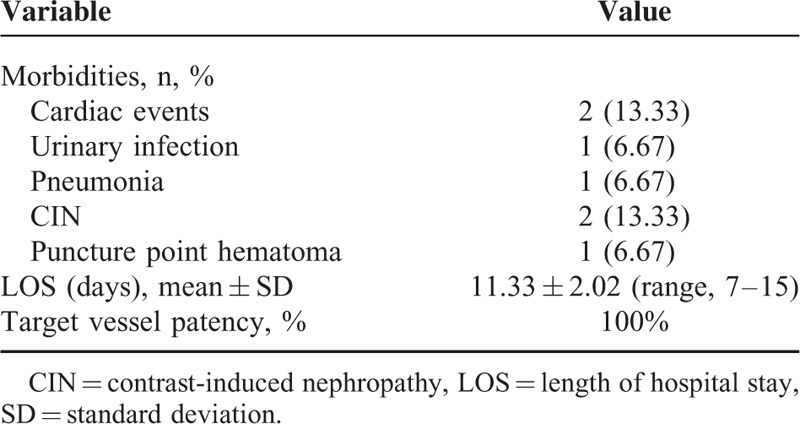
Follow-Up Data

## DISCUSSION

With rapid advancements in EVAR globally, a shift from open repair to EVAR for the treatment of AAAs was seen in China over the last decade. EVAR has several advantages over open surgery in the reduction of mortality and morbidity.^1,2,8^ However, JAAAs with an unsuitable proximal fixation zone cannot be managed with conventional EVAR owing to high incidences of proximal endoleak, device migration, and aneurysm rupture. Initial reports from single centers^9,10^ and multiple centers^5,7,11^ have suggested that FEVAR using the Zenith fenestrated device is effective for the treatment of JAAAs. Our study found that FEVAR using the Zenith fenestrated device is effective for the treatment of JAAAs in the mainland of China, with satisfactory outcomes. However, the number of FEVAR procedures using the Zenith fenestrated device is lower in China than in developed countries.^7,11^ In China, the cost of the Zenith fenestrated device is about US$50,000, and even for patients with medical insurance, the cost is high at approximately USD$40,000. Most patients are unable to afford the Zenith fenestrated device and therefore choose other treatments. Additionally, the relatively large delivery system is not appropriate for female Chinese patients. Therefore, only male patients were included in the present study. Moreover, presently, there is no generally accepted definition for JAAAs, and physicians continue to use conventional devices for the treatment of patients who might actually benefit from sophisticated devices in China.

In the present study, the fenestrated stent graft was successfully deployed in all patients, and the technical success rate was 100%. Additionally, the 30-day postoperative mortality rate was 0%, and the all-cause mortality rate was 13.33%. Moreover, no death occurred owing to artery rupture. These results are consistent with those reported from developed countries.^7,11^ The relatively strict criteria of patient selection and the meticulous manufacturing process for the device (approximately 12 weeks) based on CTA images might have been responsible for the good results.

The device implantation should be performed by physicians who have a lot of experience, and clear anatomical criteria are required for the design of these fenestrated devices. In China, only few large centers can perform FEVAR. In the present study, 4 of the 15 patients did not have standard anatomical inclusive criteria for the Zenith device. Two patients had infrarenal lengths of 4 and 6 mm, and the infrarenal angle was 60° in both patients. The other 2 patients had infrarenal angles of 30° and 0°, and the infrarenal length was 0 mm in both patients. Device migration or stent fracture was not noted in any of these 4 patients. However, 1 patient who had an infrarenal angle of 60° and infrarenal length of 4 mm experienced a type Ia endoleak.

The mean volume of contrast medium used was 303.33 ± 56.27 mL (range, 250–450 mL) which was much more than infrarenal abdominal aneurysm endovascular repair. In this study, 2 patients with renal insufficiency underwent CIN after operation. So how to reduce contrast medium and protect renal function, especially for patients with renal insufficiency (RI) is necessary to be noted. Hydration therapy (1000 mL normal saline in the 12 hours before the operation to the 1st 24 hours after FEVAR) is an effective way to lower the risk of CIN. Compared with low-osmolality, nonionic iohexol, iso-osmolality, nonionic iodixanol such as Iodixanol (Visipaque) is more easily to decrease the incidence of CIN in patients with RI.^12^ Proper use of markers in the fenestrated stent graft, accurately preoperative anatomy landmarker positioning, longer interval between 2 contrast injections and infrequent aimless angiography can reduce contrast medium in the procedure. Intravascular ultrasound (IVUS) could be an alternative to intraarterial contrast agents (IACA). However, it is impossible to determine stent alignment and endoleaks accurately for IVUS, so the combination of IVUS and IACA is a better way to achieve good result and reduce contrast medium.^13^ Three-dimensional (3D) image fusion of a preoperative computed tomography (CT) scan with an intraoperative cone-beam computed tomography (CBCT) image can overlay important anatomical marks on the fluoroscopy screen to facilitate endovascular orientation, thereby cutting down the requirement for repeated contrast injections during procedures.^14,15^ Dijkstra et al^16^ accomplished FEVAR with this technique and had satisfactory outcome. 3D image integrated with IVUS or carbon dioxide (CO_2_) digital subtraction angiograms (DSA) could diminished as much as near 60% of contrast dose in conservative FEVAR.^17,18^

In view of an accurate detection of endoleaks with high sensitivity, CTA is a favorable method for surveillance during the follow-up period.^19,20^ Presently, there is no consensus on the optimal CT image acquisition protocol for examining a patient after endograft placement.^21^ The typical protocol includes 3 separate phases: a nonenhanced examination, an arterial phase, and a venous phase.^22^ In order to avoid mistaking calcifying thrombus having high attenuation for a endoleak on the contrast-enhanced images, noncontrast CT is essential. Type I and type III endoleaks can be generally detected during arterial phase. However, not all type II endoleaks can be visualized during the arterial phase, and even during the venous phase, since the contrast will not have had time to propagate throughout the isolated aneurysm by the time images are acquired, further, the blood backflow from the branch arteries in isolated aneurysm are highly variable and often slow,^23–25^ which makes type II endoleak hard to detect. Although delayed phase acquisition,^26^ contrast-enhanced ultrasonography (CEUS),^27^ and delayed phase MR imaging with a blood pool agent^28^ were proposed to detect low-flow endoleaks, we hold that only under the circumstance that the aneurysm sac is noted to be getting larger and no endoleak is seen, these measures could be performed. In our series, 11 patients without any detected endoleaks in our CT image acquisition protocol might develop low-flow endoleaks. In spite of this, on account of no enlargement of their aneurysm sac, further investigations were not carried out. Two type type II endoleaks were visualized not during the arterial phase but during the venous phase.

There is little debate on the prompt treatment of type I and type III endoleaks that imply inadequate exclusion of the aneurysm sac. However, in our opinion, whether to treat type I endoleak promptly depends on the degree of it. This could be judged by comparing the speed and intensity of flow of the contrast agent into the main body of stent graft and the aneurysm sac. If these are the same, the endoleak could be judged to be serious and should be dealt with immediately. If the speed of flow into the main body of stent graft is significantly faster than into the aneurysmal sac, and the opacity in the main body of stent graft is significantly more than in the aneurysmal sac, the endoleak could be judged to be mild, with a moderate endoleak falling between the 2 and could be strictly surveilled. In this study, 1 case of immediate proximal type I endoleaks was under surveillance without any management and disappeared in the 6 month CTA. The speculative reason is the formation of thrombi, which could interrupt the perigraft flow. Another reason maybe the self-expanding nature of the stentgraft, which gave rise to gradual neck remodeling.^29,30^

The present study had some limitations. The study included a small number of patients and a short follow-up period. Studies with a larger number of patients and a longer follow-up period are needed to further confirm the findings of the present study. A national database should be created in China, similar to databases in developed countries, to allow appropriate assessment of FEVAR using the Zenith fenestrated device for JAAAs. The selection of some patients in our study was not in accordance with the anatomical criteria for the Zenith fenestrated device, and this may have influenced the results. Moreover, this was a retrospective study, and therefore, prospective studies should be performed to further confirm the findings of the present study. Nonetheless, the study showed the early and mid-term results of FEVAR using the Zenith fenestrated device for JAAAs in China.

## CONCLUSION

The early and mid-term results of FEVAR using the Zenith fenestrated device were good, demonstrating that this procedure is effective for the treatment of JAAAs. With further improvements in the standard of living and changes in the treatment preferences of physicians, FEVAR might become widely popular in China.

## References

[1] Greenhalgh RM Brown LC United Kingdom EVAR Trial Investigators. Endovascular versus open repair of abdominal aortic aneurysm. *N Engl J Med* 2010; 362:1863–1871. 20382983 10.1056/NEJMoa0909305

[2] De Bruin JL Baas AF Buth J Long-term outcome of open or endovascular repair of abdominal aortic aneurysm. *N Engl J Med* 2010; 362:1881–1889. 20484396 10.1056/NEJMoa0909499

[3] Taylor SM Mills JL Fujitani RM The juxtarenal abdominal aortic aneurysm. A more common problem than previously realized? *Arch Surg* 1994; 129:734–737. 8024454 10.1001/archsurg.1994.01420310066011

[4] Park JH Chung JW Choo IW Fenestrated stent-grafts for preserving visceral arterial branches in the treatment of abdominal aortic aneurysms: preliminary experience. *J Vasc Interv Radiol* 1996; 7:819–823. 8951748 10.1016/s1051-0443(96)70854-0

[5] Greenberg RK Sternbergh WC 3rd Makaroun M Intermediate results of a United States multicenter trial of fenestrated endograft repair for juxtarenal abdominal aortic aneurysms. *J Vasc Surg* 2009; 50:730–737.e1. 19786236 10.1016/j.jvs.2009.05.051

[6] Verhoeven EL Vourliotakis G Bos WT Fenestrated stent grafting for short-necked and juxtarenal abdominal aortic aneurysm: an 8-year single-centre experience. *Eur J Vasc Endovasc Surg* 2010; 39:529–536. 20202868 10.1016/j.ejvs.2010.01.004

[7] British Society for Endovascular Therapy, the Global Collaborators on Advanced Stent-Graft Techniques for Aneurysm Repair (GLOBALSTAR) Registry. Early results of fenestrated endovascular repair of juxtarenal aortic aneurysms in the United Kingdom. *Circulation* 2012; 125:2707–2715. 22665884 10.1161/CIRCULATIONAHA.111.070334

[8] Lederle FA Freischlag JA Kyriakides TC Outcomes following endovascular vs open repair of abdominal aortic aneurysm: arandomized trial. *JAMA* 2009; 302:1535–1542. 19826022 10.1001/jama.2009.1426

[9] Tambyraja AL Fishwick NG Bown MJ Fenestrated aortic endografts for juxtarenal aortic aneurysm: medium term outcomes. *Eur J Vasc Endovasc Surg* 2011; 42:54–58. 21514856 10.1016/j.ejvs.2011.03.033

[10] Liao TH Watson JJ Mansour MA Preliminary results of Zenith Fenestrated abdominal aortic aneurysm endovascular grafts. *Am J Surg* 2014; 207:417–421. 24581767 10.1016/j.amjsurg.2013.09.015

[11] Oderich GS Greenberg RK Farber M Results of the United States multicenter prospective study evaluating the Zenith fenestrated endovascular graft for treatment of juxtarenal abdominal aortic aneurysms. *J Vasc Surg* 2014; 60:1420–1428. 25195145 10.1016/j.jvs.2014.08.061

[12] Aspelin P Aubry P Fransson SG Nephrotoxicity in high-risk patients study of iso-osmolar and low-osmolar non-ionic contrast media study investigators. Nephrotoxic effects in high-risk patients undergoing angiography. *N Engl J Med* 2003; 348:491–499. 12571256 10.1056/NEJMoa021833

[13] Hoshina K Kato M Miyahara T A retrospective study of intravascular ultrasound use in patients use in patients undergoing endovascular aneurysm repair: its usefulness and a description of the procedure. *Eur J Vasc Endovasc Surg* 2010; 40:559–563. 20739201 10.1016/j.ejvs.2010.07.018

[14] Tacher V Lin M Desgranges P Image guidance for endovascular repair of complex aortic aneurysms: comparison of two-dimensional and three-dimensional angiography and image fusion. *J Vasc Interv Radiol* 2013; 24:1698–1706. 24035418 10.1016/j.jvir.2013.07.016 PMC3888792

[15] Maurel B Hertault A Sobocinski J Techniques to reduce radiation and contrast volume during EVAR. *J Cardiovasc Surg (Torino)* 2014; 55 (2 suppl 1):123–131. 24796905

[16] Dijkstra ML Eaqleton MJ Greenberg RK Intraoperative C-arm cone-beam computed tomography in fenestrated/branched aortic endografting. *J Vasc Surg* 2011; 53:580–590. 10.1016/j.jvs.2010.09.039 21129898

[17] McNally MM Scali ST Feezor RJ Three-dimensional fusion computed tomography decreases radiation exposure, procedure time, and contrast use during fenestrated endovascular aortic repair. *J Vasc Surg* 2015; 61:309–316. 25175634 10.1016/j.jvs.2014.07.097 PMC4308450

[18] Koutouzi G Henrikson O Roos H EVAR guided by 3D image fusion and CO2 DSA: a new imaging combination for patients with renal insufficiency. *J Endovasc Ther* 2015; 22:912–917. 26384396 10.1177/1526602815605468

[19] Baum RA Stavropoulos SW Carpenter JP Endoleaks after endovascular repair of abdominal aortic aneurysms. *J Vasc Interv Radiol* 2003; 14:1111–1117. 14514802 10.1097/01.rvi.0000085773.71254.86

[20] Veith FJ Baum RA Ohki T Nature and significance of endoleaks and endotension:summary of opinions expressed at an international conference. *J Vasc Surg* 2002; 35:1029–1035. 12021724 10.1067/mva.2002.123095

[21] Stavropoulos SW Charagundla SR Imaging techniques for detection and management of endoleaks after endovascular aortic aneurysm repair. *Radiology* 2007; 243:641–655. 17517926 10.1148/radiol.2433051649

[22] Rozenblit AM Patlas M Rosenbaum AT Detection of endoleaks after endovascular repair of abdominal aortic aneurysm: value of unenhanced and delayed acquisitions. *Radiology* 2003; 227:426–433. 12676973 10.1148/radiol.2272020555

[23] Clough RE Carrell TW Uribe S Flow-sensitised dynamic magnetic resonance imaging (MRI) can identify dominant false lumen flow and secondary entry tears in type B aortic dissection: implications for endovascular treatment. *Br J Sur* 2011; 98:1–15.

[24] Karmonik C Bismuth J Redel T Impact of tear location on hemodynamics in a type B aortic dissection investigated with computational fluid dynamics. *Conf Proc IEEE Eng Med Biol Soc* 2010; 2010:3138–3141. 21096590 10.1109/IEMBS.2010.5627193

[25] Cheng Z Tan FP Riga CV Analysis of flow patterns in a patient-specific aortic dissection model. *J Biomech Eng* 2010; 132:051007. 20459208 10.1115/1.4000964

[26] Iezzi R Cotroneo AR Filippone A Multidetector-row computed tomography angiography in abdominal aortic aneurysm treated with endovascular repair: evaluation of optimal timing of delayed phase imaging for the detection of low-flow endoleaks. *J Comput Assist Tomoqr* 2008; 32:609–615. 10.1097/RCT.0b013e31814b271d 18664850

[27] Golzarian J Delayed helical CT acquisition in the detection of endoleak. *Radiology* 2004; 230:299–300. 14695403 10.1148/radiol.2301030837

[28] Clough RE Hussain T Uribe S A new method for quantification of false lumen thrombosis in aortic dissection using magnetic resonance imaging and a blood pool contrast agent. *J Vasc Surg* 2011; 54:1251–1258. 21906904 10.1016/j.jvs.2011.05.022

[29] Wilderman M Sanchez LA Fenestrated grafts or debranching procedures for complex abdominal aortic aneurysms. *Perspect Vasc Surg Endovasc Ther* 2009; 21:13–18. 19129204 10.1177/1531003508330477

[30] Hiramoto JS Commentary: multiple chimney grafts for total endovascular revascularization of the visceral arteries in the setting of ruptured TAAA: inventive but let's wait for the smoke to clear on this one. *J Endovascualr Ther* 2010; 17:222–223. 10.1583/09-2925C1.1 PMC2861406 20426643

